# *Akkermansia muciniphila*-induced trained immune phenotype increases bacterial intracellular survival and attenuates inflammation

**DOI:** 10.1038/s42003-024-05867-6

**Published:** 2024-02-16

**Authors:** Ainize Peña-Cearra, Ainhoa Palacios, Aize Pellon, Janire Castelo, Samuel Tanner Pasco, Iratxe Seoane, Diego Barriales, Jose Ezequiel Martin, Miguel Ángel Pascual-Itoiz, Monika Gonzalez-Lopez, Itziar Martín-Ruiz, Nuria Macías-Cámara, Naiara Gutiez, Sarai Araujo-Aris, Ana Mª Aransay, Héctor Rodríguez, Juan Anguita, Leticia Abecia

**Affiliations:** 1 Inflammation and Macrophage Plasticity Laboratory, CIC bioGUNE-BRTA (Basque Research and Technology Alliance), Derio, Spain; 2 Department of Immunology, Microbiology and Parasitology, Faculty of Medicine and Nursery, University of the Basque Country, Bilbao, Spain; 3 Genome Analysis Platform, CIC bioGUNE-BRTA (Basque Research and Technology Alliance), Derio, Spain; 4 CIBERehd, Instituto de Salud Carlos III, Madrid, Spain; 5 Ikerbasque, Basque Foundation for Science, Bilbao, Spain; 6 Present Address: Centre for Host-Microbiome Interactions, Faculty of Dentistry, Oral and Craniofacial Sciences, King’s College London, London, United Kingdom; 7 Present Address: Cell Therapy, Stem Cells and Tissues Group, CVTTH/Biobizkaia Health Research Institute, Galdakao, Spain

**Keywords:** Bacterial host response, Microbiota

## Abstract

The initial exposure to pathogens and commensals confers innate immune cells the capacity to respond distinctively upon a second stimulus. This training capacity might play key functions in developing an adequate innate immune response to the continuous exposure to bacteria. However, the mechanisms involved in induction of trained immunity by commensals remain mostly unexplored. *A. muciniphila* represents an attractive candidate to study the promotion of these long-term responses. Here, we show that priming of macrophages with live *A. muciniphila* enhances bacterial intracellular survival and decreases the release of pro- and anti-inflammatory signals, lowering the production of TNF and IL-10. Global transcriptional analysis of macrophages after a secondary exposure to the bacteria showed the transcriptional rearrangement underpinning the phenotype observed compared to acutely exposed cells, with the increased expression of genes related to phagocytic capacity and those involved in the metabolic adjustment conducing to innate immune training. Accordingly, key genes related to bacterial killing and pro-inflammatory pathways were downregulated. These data demonstrate the importance of specific bacterial members in the modulation of local long-term innate immune responses, broadening our knowledge of the association between gut microbiome commensals and trained immunity as well as the anti-inflammatory probiotic potential of *A. muciniphila*.

## Abstract (web summary)

Priming macrophages with *A. muciniphila* enhances intracellular survival and diminishes pro- and anti-inflammatory signals. Transcriptional analysis of macrophages shows upregulation of genes related to phagocytic capacity and metabolic adjustments.

## Introduction

The interaction between microbial populations or single species and the host innate immune system is poorly understood, although a few mechanisms of microbe-driven immunomodulation have already been described^1^. The intestine contains high frequencies of macrophages, which use phagocytic processes to sample their environment and help discriminate endogenous from foreign antigens^2^. This includes interactions with the resident microbiota and their components, which are required for the production of regulatory cytokines by intestinal macrophages, such as IL-1β and IL-10, the latter being directly induced by microbial species^3,4^ or their metabolic byproducts^5^. Moreover, the presence of bacteria residing intracellularly^6^ or the microbial induction of long-term responses in mononuclear cells has been suggested as other mechanisms by which microbes can modulate innate immune responses^6,7^. Recently, our group showed that previous contact with the bacterial commensal *Lactiplantibacillus plantarum* reprograms the transcriptional and metabolic profiles of innate immune cells (monocytes/macrophages) in the long term, leading to enhanced bacterial intracellular survival and decreased pro-inflammatory features^8^. However, more studies are needed to delve into the potential impact of permanent members of the intestinal commensal microbiota in the development of these prolonged responses^9^, as well as to ascertain their role in host immune homeostasis.

Among the members of the normal intestinal microbiota, *Akkermansia muciniphila* has attracted a growing interest due to its health-promoting effects in animals and humans^10^ and the alterations found in its abundance in several diseases^11^, and it is now being considered a “next-generation beneficial microbe”^12^. This abundant, mucin-degrading bacterium plays a major role in regulating host biological functions, including maintaining homeostasis^13,14^ and reinforcing the intestinal barrier^15^. Its probiotic properties have been widely studied in metabolic disorders and intestinal inflammation^12^. Although heat- or pressure-killing the bacteria completely abolishes its protective effects against metabolic syndrome in mice^15^, pasteurized *Akkermansia* is more efficient than live bacteria in a mouse model of diet-induced obesity^16^ and has proven safety and efficacy in different murine studies and in a proof-of-concept study in humans^15,17^. Therefore, despite pasteurization, it still improves host health and is recognized as a new food by the European Union^18^. *A. muciniphila*’s exposed cell components, such as Amuc_1100 protein and a specific cell membrane phospholipid, partially recapitulate its immunomodulatory activity, by signaling through Toll-like-receptor (TLR) 2/4 and TLR 2/1 heterodimers, respectively^19,20^. Additionally, *A. muciniphila*-produced ornithine lipids can negatively regulate host inflammation^21^. However, other immunomodulatory mechanisms that might require bacterial viability have been recently described, such as *A. muciniphila*-secreted threonyl-tRNA synthetase, which modulates immune homeostasis and attenuates colitis in mice by targeting macrophage polarization^22^. Hence, viable and killed *A. muciniphila*’s immunomodulatory singularities still remain to be fully explored.

To understand the interactions between *A. muciniphila* and the host innate immune cells, we analyzed macrophage responses to this species by transcriptomic analyses, as well as its ability to survive within innate immune cells and to promote the development of long-term trained immunity. In addition, we studied the impact of *A. muciniphila* on the inflamed intestinal tissue, deepening the immunomodulatory capacity of this microbe.

## Results

### *A. muciniphila* is able to survive intracellularly within phagocytic cells

**Fig. 1:**
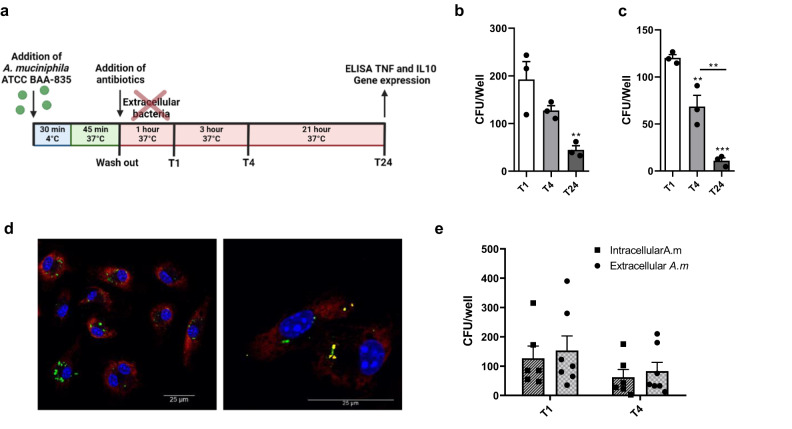
*Akkermansia muciniphila* colonizes and survives within bone marrow macrophages (BMMs) and human monocytes (hmon). **a** Antibiotic-protection assay set up. Gene expression analysis and cytokine release quantification were carried out 24 h after stopping *A. muciniphila* phagocytosis by BMMs or hmon. *A. muciniphila* intracellular survival in **b** BMMs and **c** hmon measured at T1, T4, and T24, expressed as CFU/well (*n* = 3). **d** Detection of live *A. muciniphila* inside BMMs by Live/Dead staining at T4. Live *A. muciniphila* is shown in green, and heat-killed *A. muciniphila* in yellow (Scale bar: 25 μm) (*n* = 6 for intracellular, *n* = 7 for extracellular). **e** Intracellular and extracellular *A. muciniphila* (CFU/Well) after BMMs colonization. Data are shown as mean ± SEM. Statistical analysis: **b**, **c** Ordinary one-way ANOVA (Tukey post hoc), **d** two-way ANOVA (Sidak post hoc).

Previous reports have shown the presence of bacterial species residing inside immune cells in mesenteric lymph nodes, having an impact on the modulation of immune responses^23^. To unveil the potential of *A. muciniphila* to survive inside phagocytes, we first used in vitro antibiotic-protection assays (Fig. 1a). Bacteria were added to cells, allowed to attach at low temperatures, and incubated at physiological conditions to facilitate internalization. Cells were then washed, and fresh media containing antibiotics was added. After 1, 4, or 24 h, phagocytes were lysed, and suspensions were plated on agar to quantify intracellular bacteria. Remarkably, *A. muciniphila* was able to survive intracellularly both in bone marrow-derived macrophages (BMMs) (Fig. 1b) and human monocytes (Fig. 1c) for up to 24 h. Indeed, the presence of live bacteria inside macrophages was further confirmed by using Live/Dead staining and confocal microscopy (Fig. 1d). Since we observed that *A. muciniphila* was able to survive within phagocytes, we wondered whether it would also be able to escape from these cells. Therefore, after phagocytosis of bacteria, macrophages were left for either 1 or 4 h in a medium supplemented with antibiotics to eliminate the remaining extracellular bacteria. BMMs were then washed and further incubated for 24 h in an antibiotic-free medium. Remarkably, similar numbers of viable bacteria were detected in the intracellular and extracellular compartments (Fig. 1e), showing *A. muciniphila* capacity to escape from macrophages. Taken together, these findings manifest the ability of *A. muciniphila* to colonize, survive, and escape from phagocytes.

### Live *A. muciniphila* is a potent immunomodulator of macrophage responses

**Fig. 2:**
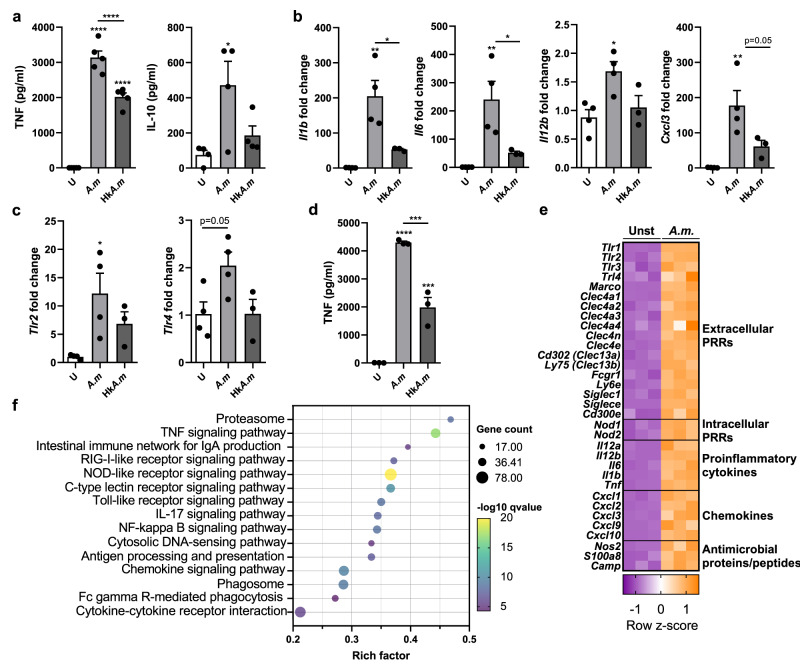
*A. muciniphila* modulates the response of murine macrophages and human monocytes. **a** TNF and IL-10 measurements in BMMs stimulated for 24 h with live (*A.m*) or heat-killed (Hk*A.m*) *A. muciniphila* by ELISA (*n* = 5 for TNF, *n* = 4 for IL-10). **b**, **c** Gene expression levels in stimulated BMMs expressed relatively to the mean of unstimulated BMMs (U), analyzed by Real-Time PCR (*n* ≥ 3). **d** TNF quantification by ELISA in human monocyte stimulated supernatants (*n* = 3). **e** Heatmap of gene expression for several differentially expressed genes between live *A.m*-stimulated and unstimulated BMMs, analyzed by RNAseq (*n* = 3). Normalized expression values are z score normalized for each gene. **f** Bubble plot displaying significantly enriched KEGG pathways of upregulated genes in acutely stimulated BMMs. Only relevant pathways related to immune processes are selected and represented here, extracted from the Supplementary data set 1. Rich factor is calculated as the number of DEGs associated with each pathway by the total number of genes belonging to that pathway in KEGG database. Data are shown as mean ± SEM. Statistical analysis: Ordinary one-way ANOVA (Tukey post hoc).

To delve into the activation status of BMMs when acutely exposed to either live (*A.m*) or heat-killed (Hk*A.m*) *A. muciniphila*, TNF and IL-10 levels were determined by ELISA. The release of TNF and IL-10 exhibited their highest levels when BMMs were co-incubated with live bacteria compared to Hk*A.m* (*p* = 0.0001 for TNF), although it wasn´t significant in the case of IL-10 production (Fig. 2a). Moreover, while the pro-inflammatory cytokine- and chemokine-coding genes *Il1b* (*p* = 0.0021), *Il6* (*p* = 0.0068), *Il12b* (*p* = 0.0314) and *Cxcl3* (*p* = 0.0045) were highly upregulated after live *A. muciniphila* encounter, stimulation with heat-killed bacteria resulted in a more modest or no upregulation of these genes (Fig. 2b). In addition, gene expression of the Toll-like receptors (TLRs) *Tlr2* and *Tlr4* was also increased in response to stimulation (*p* = 0.0260 for *Tlr2* and *p* = 0.0512 for *Tlr4*) with live but not with heat-killed bacteria (Fig. 2c). The same functional phenotype was observed in human CD14^+^ monocytes acutely stimulated with live bacteria, with a significant increase in TNF production compared to cells exposed to heat-killed *A. muciniphila* (*p* = 0.0005) (Fig. 2d). These results suggest that the acute stimulation of naïve monocytes/macrophages induces strong cytokine responses, particularly when cells are stimulated with live *A. muciniphila*.

Given the higher immunostimulatory potential of viable *A. muciniphila* compared to heat-killed, we sought to assess the transcriptional traits underlying macrophage responses to this commensal bacterium in its live form. We, therefore, analyzed the transcriptome of BMMs acutely stimulated for 16 h with live *A. muciniphila* by RNA sequencing. We found a great number of differentially expressed genes (Supplementary Fig. 1a) when cells were stimulated, with a clear transcriptional signature derived from stimulation (Supplementary Fig. 1b). Pattern-recognition receptors (PRRs) found to be strongly upregulated included not only TLRs already described to be involved in *A. muciniphila* recognition (*Tlr1*, *Tlr2*, *Tlr4*), but also NOD-like receptors (*Nod1* and *Nod2*), C-type lectin receptors (mostly members of the C-type lectin domain family 4) and the scavenger receptor, *Marco*, among others (Fig. 2e). These cell-surface and intracellular receptors could represent novel proteins likely to be involved in the sensing and internalization of *A. muciniphila* by macrophages. The cascade of events activated upon the engagement of multiple receptors led to the robust increase of transcripts encoding for pro-inflammatory cytokines (*Il12b*, *Il12a*, *Il1b*, *Il6*, *Il1a*, members of the TNF superfamily) and chemokines (*Cxcl1, 2, 3, 9 10*), along with several potential antibacterial proteins/peptides coding genes such as nitric oxide synthase 2 (*Nos2*), calgranulin A (*S100a8*) and cathelicidin antimicrobial peptide (*Camp*) (Fig. 2e). Additionally, functional enrichment analysis based on KEGG pathways of the significantly upregulated genes confirmed the induction of pathways involving the aforementioned receptors, as well as a general activation status of phagocytes. These pathways include NOD-, Toll- and C-type lectin receptor, FcgR signaling, TNF signaling, phagosome, *Il17* and NF-kappa B signaling pathway, chemokine signaling pathway, and antigen processing and presentation, among others (Fig. 2f, Supplementary data set 1), and manifest the potent immunostimulatory capacity of live *A. muciniphila* on naïve macrophages.

### *A. muciniphila* promotes an anti-inflammatory phenotype in activated intestinal macrophages

**Fig. 3:**
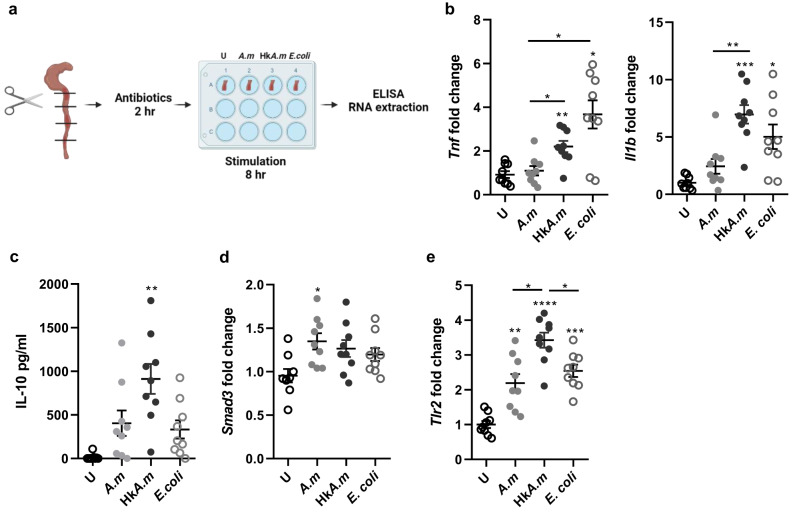
Stimulation of murine-inflamed colons with live *Akkermansia muciniphila* promoted an anti-inflammatory response. **a** Diagram of the steps to prepare murine colon tissues and stimulate ex vivo with live *A.m*, heat-killed *A.m,* and *E. coli*. Gene expression levels in all conditions of **b** *Tnf* and *Il1b*, and **d** *Smad3* and **e** *Tlr2* shown as fold change relative to the mean of unstimulated colon tissue (U) (*n* = 9). **c** IL-10 quantification in medium supernatants by ELISA (*n* = 9). Data are represented as mean ± SEM. Statistical analysis: One-way ANOVA (Tukey post hoc) and mixed-effects analysis for paired samples (Tukey post hoc). **a** Created with BioRender.com.

To assess the potential immunoregulatory role of *A. muciniphila* on activated phagocytes, such as those found during intestinal inflammation, colon sections of mice with induced colitis, which exhibit an increased tissue infiltration of activated macrophages, were harvested, partitioned, and treated with antibiotics. Colon sections were then stimulated with either live (*A.m*) or heat-killed (Hk*A.m*) *A. muciniphila*, pathogenic *E. coli* (as a control of a pro-inflammatory bacteria) or left unstimulated (U) (Fig. 3a). The stimulation of inflamed colon sections with live *A. muciniphila* did not induce changes in either *Tnf* or *Il1b* gene expression levels compared to unstimulated controls. In turn, tissues exposed to heat-killed *A. muciniphila* or pathogenic *E. coli* showed increased expression levels of both genes (*p* < 0.05) (Fig. 3b). In addition, it was determined that the colons of healthy mice, upon exposure to different bacteria, failed to elicit inflammatory response. IL-10 release, measured by ELISA, showed higher levels only when the tissue was stimulated with heat-killed *A. muciniphila* (*p* = 0.0035) (Fig. 3c). In this sense, the expression of *Smad3*, an intracellular signaling protein in the canonical TGF-β pathway, was also elevated (*p* = 0.0352) in tissue sections exposed to live *A. muciniphila*, confirming the anti-inflammatory effect of the bacterium under inflammatory conditions in the colon tissue, with no significant differences being found with the other two stimuli used (Fig. 3d). In addition, *Tlr2* expression was promoted after the stimulation with live bacteria, although at lower expression levels compared to heat-killed *A. muciniphila* (*p* = 0.0221) (Fig. 3e). These data support the modulatory effects of *A. muciniphila* on activated macrophages in an experimental colitis model through the reduction of inflammatory cytokines and the increase in IL-10 production, as well as the capacity to signal in response to TGF-β.

### Pre-exposure of macrophages to *A. muciniphila* enhances bacterial survival and decreases inflammation

Our results showed that, in contrast with the ex vivo stimulation of pre-activated phagocytes within inflamed colonic tissue, the acute stimulation of naïve monocytes/macrophages results in a pro-inflammatory phenotype, particularly when cells were stimulated with live *A. muciniphila*. The modulation of the pro-inflammatory response observed ex vivo might be derived from the persistent contact of commensal microorganisms, such as *A. muciniphila*, with intestinal innate immune cells, thereby altering the response of these cells to the bacterium. This led us to analyze the plasticity of macrophages after interacting with *A. muciniphila* through mechanisms related to trained immunity.

**Fig. 4:**
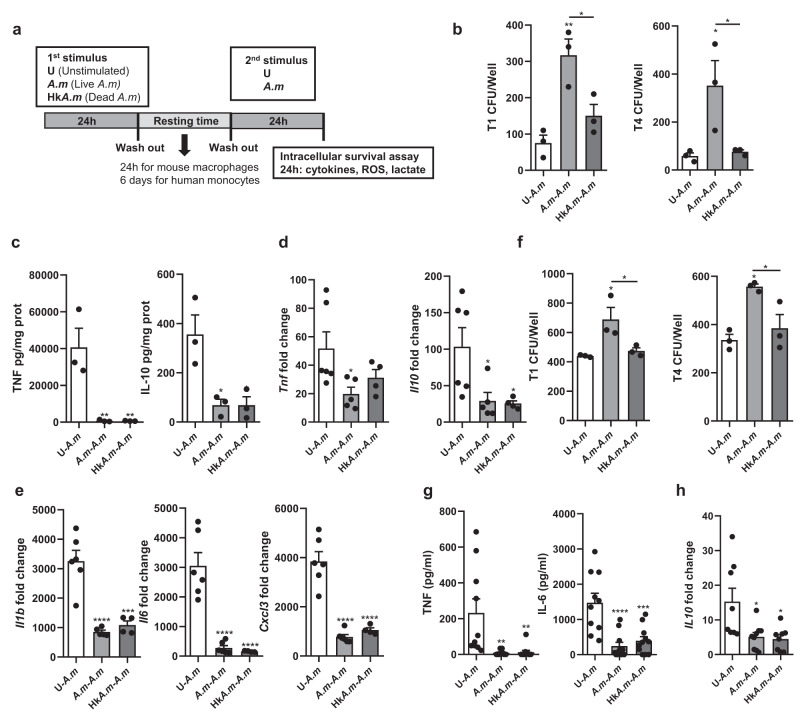
BMMs and human monocyte training with live *Akkermansia muciniphila* enhances its intracellular survival capacity and reduces the pro-inflammatory response. **a** Experimental workflow of in vitro trained innate immunity assay. **b** Intracellular survival of *A. muciniphila* in acute (U-*A.m*) or trained (*A.m-A.m*, Hk*A.m*-*A.m*) BMMs at T1 and T4 represented as CFU/Well (*n* = 3). **c** TNF and IL-10 quantification in BMMs supernatants (*n* = 3). Gene expression levels in BMMs of **d** *Tnf* and *Il-10* and **e** *Il-1b*, *Il-6*, and *Cxcl3* (*n* ≥ 4). **f** *A. muciniphila* intracellular survival within acute and trained monocytes at T1 and T4 after internalization (*n* = 3). **g** TNF and IL-6 quantification by ELISA in human monocytes supernatants (*n* ≥ 10). **h** *IL10* expression of human monocytes (*n* ≥ 8). qPCR results are normalized to the unstimulated control (U-U), not shown in the graphs. Data are shown as mean ± SEM, *n* = 3–6. Statistical analysis: ordinary one-way ANOVA (Tukey post hoc).

To investigate this, phagocytes were stimulated for 24 h with live or heat-killed *A. muciniphila*, rested for 24 hours, and then restimulated with live bacteria. Unstimulated cells at both time points serve as controls (Fig. 4a). Pre-stimulation of BMMs in vitro with live *A. muciniphila* (*A.m*-*A.m*) enhanced bacterial intracellular survival in a second encounter in comparison with previously unstimulated cells (U-*A.m*) at early time points (T1 (*p* = 0.0057) and T4 (*p* = 0.0329)) (Fig. 4b), while no significant differences were observed after 24 h. Of note, no bacterial growth was observed when macrophages were lysed after the resting period, showing that bacteria did not continue surviving inside BMMs. However, priming macrophages with heat-killed *A. muciniphila* (Hk*A.m-A.m*) failed to recapitulate the enhanced bacterial survival elicited by live bacteria. The inflammatory output of macrophages was further reshaped (in the long term) by innate immune training after a period of resting. Pre-stimulation with either live or heat-killed bacteria induced a substantial reduction in TNF and IL-10 production in a second encounter compared to acute stimulation, both at the protein (*p* ≤ 0.01) (Fig. 4c) and gene expression levels (*p* ≤ 0.01) (Fig. 4d). Additionally, *Il1b*, *Il6,* and *Cxcl3* also presented a pronounced decrease in gene expression in both groups of primed macrophages compared to acutely stimulated cells (*p* < 0.05) (Fig. 4e).

These changes in innate immune cell behavior induced by *A. muciniphila* were confirmed in human monocytes. Indeed, *A. muciniphila* also presented a higher capacity of intracellular survival within human monocytes primed with live *A. muciniphila* 1 and 4 h after internalization, compared to both acutely stimulated monocytes (*p* ≤ 0.02) and those primed with heat-killed bacteria (*p* < 0.05) (Fig. 4f). Upon priming, monocytes not only produced diminished levels of the pro-inflammatory cytokines TNF and IL-6 (*p* = 0.001) (Fig. 4g), but also presented lower *IL10* gene expression levels compared to the U-*A.m* group (*p* = 0.01) (Fig. 4h).

These data indicate that the exposure to live *A. muciniphila* modulates the long-term phenotype of phagocytic cells. While both live and heat-inactivated forms of *A. muciniphila* induce a similar secondary response in terms of cytokine output, the viability of the bacterium during the training span of phagocytes seems to be essential for increased bacterial survival in a subsequent encounter. This might underlie the existence of different pathways for training induction related to inflammation and intracellular survival.

### Transcriptional and metabolic traits underlying long-term macrophage responses to live *A. muciniphila*

**Fig. 5:**
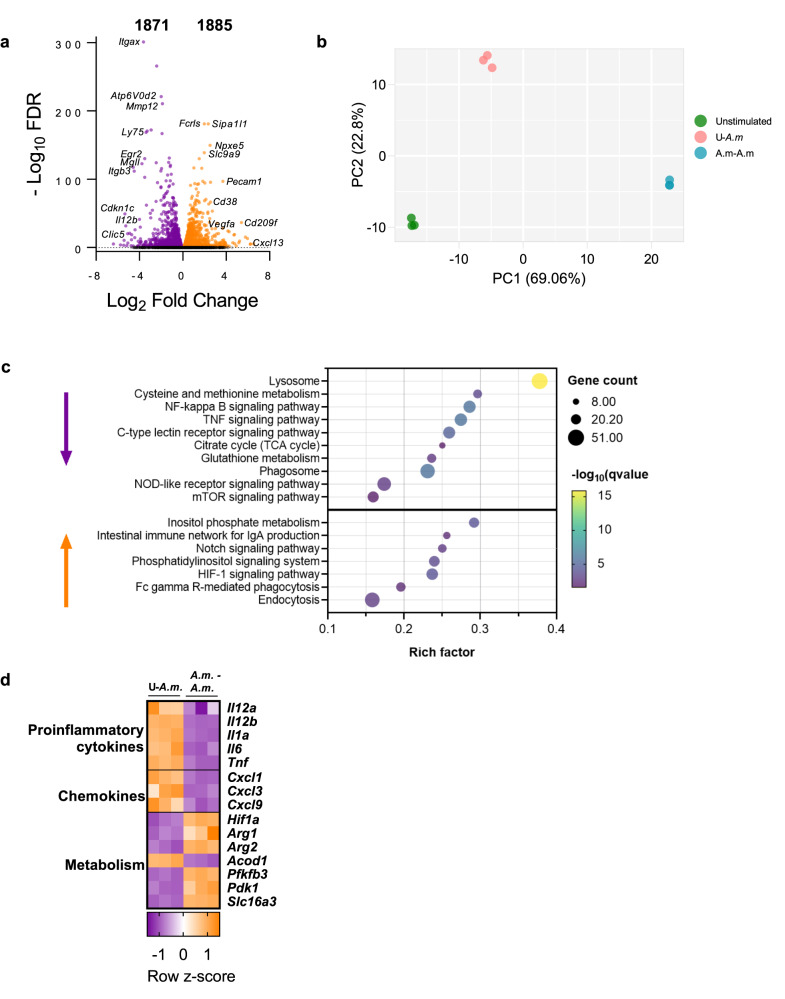
Transcriptional response of live *Akkermansia muciniphila*-trained BMMs. **a** Volcano plot representing differentially expressed genes of trained and restimulated macrophages compared to acutely stimulated cells. **b** Principal component analysis (PCA) of the transcriptome of trained, acute, and unstimulated macrophages (*n* = 3). **c** Bubble plot of significantly enriched KEGG pathways of upregulated and downregulated genes in trained macrophages. Only relevant pathways related to immune and metabolic processes are selected and displayed here, extracted from the Supplementary data set 2. **d** Heatmap of gene expression for several differentially expressed genes between live *A. muciniphila* acutely stimulated and trained BMMs. Normalized expression values are *z* score normalized for each gene.

To determine the mechanisms involved in these long-term innate responses to live *A. muciniphila*, we further studied the transcriptional profile of trained BMMs. Trained macrophages showed a clear distinct transcriptional profile compared to those acutely stimulated and unstimulated controls (Fig. 5a) with a sizeable number of differentially expressed genes between acutely and restimulated phagocytes (Fig. 5b). Consistent with our previous findings, KEGG pathway analysis uncovered NF-kappa B signaling and TNF signaling pathways as strongly downregulated in trained macrophages (Fig. 5c), along with the downregulation of several pro-inflammatory cytokines *(Il12a, Il12b, Il1a*, *Il6*, *Tnf*) and chemokines (*Cxcl1, Cxcl3, Cxcl9*) (Fig. 5d). Importantly, pathway analysis also revealed lysosome as the most significantly downregulated function in trained phagocytes. This pathway, together with downregulated phagosomal function, could potentially account for the increased intracellular survival of *A. muciniphila* observed during a second encounter. Moreover, the upregulation of Fc gamma-receptor-mediated phagocytosis might be associated with the enhanced internalization of *A. muciniphila* in macrophages previously stimulated with live bacteria. In addition, *Akkermansia* upregulates the IgA production signaling pathway in trained macrophages, suggesting that it may contribute to a reduced inflammatory response. It is noteworthy that the upregulation of *Arg1* and *Arg2*, encoding the two isoforms of the enzyme arginase indicates immune suppression in macrophages.

**Fig. 6:**
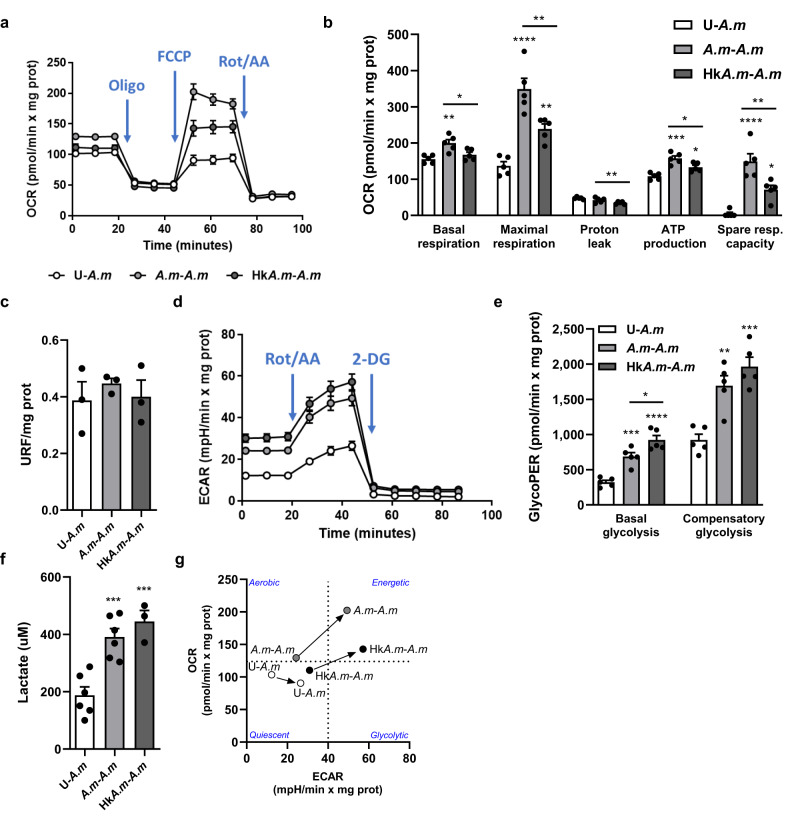
*A. muciniphila*-training of BMMs promotes a profound metabolic rewiring towards glycolysis and mitochondrial respiration. **a**, **b** Seahorse Extracellular Flux Analyzer was used to determine oxygen consumption rate (OCR) coupled to mitochondrial respiration (*n* = 5). **c** Intracellular ROS superoxide production in the mitochondrial matrix of BMMs (*n* = 3, independent experiment). **d**, **e** Extracellular acidification rate (ECAR) and glycolytic parameters measured by Seahorse (*n* = 5). **f** Lactate concentration (μM) in BMM supernatants (*n* = 4–6). **g** Metabolic phenogram showing OCR/ECAR ratio in basal and stressed conditions. Data are shown as mean ± SEM. Statistical analysis: ordinary one-way ANOVA (Tukey post hoc).

HIF-1 signaling pathway was found to be one of the most significantly enriched pathways among the upregulated genes in trained macrophages (Fig. 5c). The upregulation of this pathway, frequently reported as a hallmark of metabolism reprogramming in trained innate immune cells^24^, encouraged us to examine whether priming of BMMs with *A. muciniphila* modulated their cell metabolism. Seahorse Extracellular Flux assays unveiled that training with both *A.m* and Hk*A.m* triggered a substantial metabolic rewiring characterized by increased oxygen consumption rate (OCR), which quantifies mitochondrial respiration (Fig. 6a, b). Notably, live *A.m* exhibited a more pronounced induction of this metabolic shift (Fig. 6a, b). However, reactive oxygen species (ROS) production, usually derived from the mitochondrial electron chain, was not affected (Fig. 6c). In addition to respiration, extracellular acidification rate (ECAR) and, therefore, basal and compensatory glycolysis levels were also highly induced in *A.m*. and Hk*A*.*m*.-trained cells upon the second challenge (Fig. 6d, e). Augmented glycolysis correlated with increased levels of lactate in trained cell supernatants (*A.m*-*A.m* and HkA*.m*-*A.m*) (*p* ≤ 0.001) (Fig. 6f), and with increased expression of *Pfkfb3*, the gene encoding the positive regulator of glycolysis fructose-2,6-biphosphatase. Furthermore, trained macrophages exhibited enhanced expression of pyruvate dehydrogenase (*Pdk1*) and *Slc16a3* (Fig. 5d). PDK1 is a key regulatory enzyme for glucose metabolism, blocking the conversion of pyruvate to acetyl-coenzyme A, thereby preventing pyruvate from entering the tricarboxylic acid (TCA) cycle and likely facilitating its conversion to lactate. *Slc16a3*, coding for monocarboxylate transporter 4 (MCT4), is responsible for lactate export outside the cells. In summary, Hk*A.m.-*trained macrophages, and more pronouncedly *A.m*.-trained macrophages, displayed elevated energy levels both at baseline and especially under stress conditions. This distinct energetic profile is shown in the phenogram, wherein the trained groups are clearly separated from their acute counterparts (Fig. 6g). Decreased expression of *Acod1*, coding for aconitate decarboxylase, could also suggest a reduction in the pathway involved in the production of the antimicrobial metabolite itaconate, which has also been linked to the inhibition of glycolysis at multiple levels. Other metabolic changes presumably occurring in trained macrophages, as denoted by KEGG analysis, involve upregulated inositol phosphate metabolism and downregulated cysteine and methionine metabolism, carbon metabolism, glutathione metabolism and amino sugar and nucleotide sugar metabolism (Fig. 5c, Supplementary data set 2).

Taken together, these data show that *A. muciniphila* induces trained immune responses in both murine macrophages and human monocytes, promoting a metabolic rewiring towards increased glycolysis and mitochondrial respiration and decreased cytokine production.

## Discussion

Co-evolution with the symbionts inhabiting a host includes the development of tolerance mechanisms responsible for maintaining homeostatic immune responses. This is of particular relevance at gut mucosal surfaces, which host the greatest and most diverse microbial populations^25^. While the adaptive immune system plays a paramount role in maintaining gut homeostasis^26^, intestinal innate immune cells serve essential functions, such as luminal sampling or the regulation of the gut inflammatory profile^1^. Gastrointestinal macrophages play defensive and regulatory roles both at steady state and during inflammation, encountering live microbes and microbial-derived components. These cells produce the anti-inflammatory cytokine IL-10 to maintain gut homeostasis. Mice lacking IL-10 signaling develop IBD due to excessive microbiota-derived TLR/MyD88-dependent responses and uncontrolled activation^27,28^. Furthermore, the characterization of trained immune responses^7^ presents a different paradigm through which to view host-microbe interactions. Therefore, fully understanding the microbiota-driven modulation of innate immune responses in the gut is one of the main current challenges in host-microbiota research, both in the steady state and in the context of disease.

*Akkermansia muciniphila* shows an outstanding potential for regulating gut function. Gastrointestinal decrease of abundancies of this species correlates with several pathologies^10,11^, and *A. muciniphila* has been shown to exert beneficial effects during the progression of colitis in IBD patients^29,30^. Additionally, in patients with metabolic diseases, interventions containing this microbe reduced pro-inflammatory profiles and improved intestinal barrier regulation^31^. However, little is known about the specific interactions between *A. muciniphila* (live or heat-killed), and the immune system (either in healthy or inflamed stages), and further knowledge would help improve the probiotic and immunomodulatory activities of this species.

We first explored the interactions of *A. muciniphila* with naïve macrophages and monocytes, both from murine and human origin. Stimulation with live bacteria led to the activation of pro-inflammatory responses, as previously observed for *A. muciniphila*^32^, as well as to the overexpression of a broad repertoire of activating and phagocytic pattern recognition receptors. The heat-killed form of the same microorganism, however, presented reduced immunomodulatory capacity in phagocytes. We also observed that *A. muciniphila* survives intracellularly, with a substantial bacterial population being present inside cells even 24 hours after the challenge. While intracellular survival has been largely analyzed in the context of bacterial pathogenicity, little is known of its importance when studying beneficial microbes. Of note, a recent study showed that some microbiota species reside within gastrointestinal, immune cells, and their presence is directly linked to their immunomodulatory capacity^6^, suggesting that *A. muciniphila* survival could also exploit these mechanisms.

Previous research has indicated that *A. muciniphila* ameliorates DSS-induced ulcerative colitis either by improving the microbial community or by microbe-host interactions, protecting the gut barrier function and reducing the levels of inflammatory cytokines^30^. Pasteurized *A. muciniphila* and outer membrane protein Amuc_1100 decrease mRNA levels of pro-inflammatory cytokines in mice with DSS-induced colitis^19^. Using an ex vivo stimulation model to analyze the impact of *A. muciniphila* on DSS-inflamed colon tissue, we did not observe any changes in *Tnf* or *Il1b* expression when live bacteria were used compared to unstimulated controls, while increased levels were observed after heat-killed *A. muciniphila* or *E. coli* stimulation. Of note, IL-10 release increased in all conditions, suggesting that, together with the lack of *Tnf* expression, live *A. muciniphila* induced an anti-inflammatory profile in inflamed tissues. In agreement with our results, it was reported that *Akkermansia* was positively associated with IL-10 and negatively associated with IL-6 and TNF^30^. Additionally, *Tlr2* and *Smad3* expression were also increased, with the latter only induced in the presence of live *A. muciniphila*, suggesting the involvement of these receptors in regulating responses to this species and possibly a role of the TGF-β pathway in agreement with previous studies^33^.

In recent years, a greater body of research has shown the ability of innate immune cells to develop long-term responses when exposed to primary challenges using pathogen-associated molecular patterns (PAMPs), such as LPS or β-glucan, that eventually lead to an enhanced/diminished response to a secondary, heterologous insult^7^. Though this phenomenon has been mainly explored in the context of pathogenic microbes, there is scarce information about its relevance for host interactions with commensal organisms^9^. Thus, we studied the impact of macrophage priming with *A. muciniphila* on their long-term responses. Pre-stimulation of immune cells with live, but not heat-killed, bacteria induced a significant increase of *A. muciniphila* intracellular survival in the second challenge, similar to our group’s previous observations for *Lactiplantibacillus plantarum*^8^. Even though macrophage training increased metabolic activity and reduced cytokine and chemokine expression, higher intracellular survival occurred only after live *A. muciniphila* priming. This suggests that while some common stimulatory factor(s) induce inflammatory and metabolic reprogramming during priming, unique aspects of functional bacteria (such as metabolite production) contribute to enhanced intracellular survival. In this sense, it was recently reported that the administration of supernatants from *A. muciniphila* cultures, but not pasteurized *A. muciniphila*, alleviated viral pathogen-associated mortality in mice, limiting NF-κB-mediated inflammation^34^.

The increased metabolic activity of trained macrophages might prepare the cells to respond to the bacterium faster in terms of pro-inflammatory cytokine production and possibly also through enhanced phagocytosis. In agreement, FcgR-mediated phagocytosis and endocytosis were upregulated. Moreover, the first line of defense against microorganisms, “Intestinal immune network for IgA production”, was upregulated pointing to a tolerogenic phenotype. Additionally, the expression of *Acod1*, coding for aconitate decarboxylase, was decreased in trained macrophages. The antimicrobial and immunomodulatory metabolite itaconate has been associated with the modulation of β-glucan-induced trained immunity^35^, and *ACOD1* expression has shown downregulation both in β-glucan and *L. plantarum*-trained monocytes^8,35^. Furthermore, the most significantly downregulated function in trained cells was related to lysosomes suggesting that internalized bacteria were less degraded in *A. muciniphila*-trained macrophages than in their acute counterparts. Indeed, *Slc9a9-*encoded sodium-proton exchanger NHE9 showed marked increased expression. This protein has been shown to impair the maturation-associated transport of phagosomes and thus regulate bactericidal activity in macrophages^36^.

Metabolic changes in macrophages after a second encounter with *A. muciniphila*, including inositol phosphate metabolism, may likely contribute to anti-inflammatory responses and intracellular survival. The anti-inflammatory action of inositol in colon macrophages in the DSS-induced murine model may be through inhibition of nitrosative and oxidative stress^37^. Thus, *A. muciniphila* primed macrophages promote an anti-inflammatory profile, which permits intracellular survival in macrophages by regulating signals such as nitrosative, cytokines, and ROS, which activate phagosomes and kill bacteria.

Phenotypic features of *A. muciniphila*-trained macrophages, including a reduced inflammatory output and enhanced bacterial survival, resembled those previously observed for *L. plantarum*^9^. However, in stark contrast to the drop in glycolytic and respiratory rates shown for *L. plantarum*, our results show that *A. muciniphila* training promoted increased glycolysis and mitochondrial respiration in macrophages. This indicates that the mechanisms driving trained responses, even for ecologically close bacteria, seem to differ. The overall trained response to a specific bacterium is likely dictated by the engagement of multiple receptors, along with the intracellular signals generated by internalized bacteria.

In conclusion, our data show that *A. muciniphila* can survive within macrophages and monocytes, having an impact on cellular inflammatory profiles both in the short- and long-term. Remarkably, stimulation of inflamed colon tissues with live bacteria does not alter pro-inflammatory cytokine expression, in sharp contrast with its heat-killed counterpart, highlighting the necessity of *A. muciniphila* physiological activity to exert its immunomodulatory role in the gut. Collectively, our study sheds light on the host immune system-*A. muciniphila* interactions that will help further understand the importance of this microbe both in the steady state and during gut inflammation. Further research should focus on the distinct, long-term innate cell responses in the gut that could dictate bacterial survival and the inflammatory equilibrium. With increasing research correlating healthy and diseased states with specific microbiota species, analyzing microbial stability in different conditions and their interaction with innate immune cells would provide a systems view of the tightly controlled, highly complex gastrointestinal environment.

## Methods

### Ethics statements, mice housing, and human samples

All procedures and experiments involving animals were approved by the Animal Research Ethics Board of CIC bioGUNE, according to the guidelines of the European Union Council (Directive 2010/63/EU) and Spanish Government regulations (RD 53/2013). Mice were maintained under specific pathogen-free conditions in a temperature-controlled room (23 ± 1 °C) with a 12/12-hour light/dark cycle and fed *ad libitum*. Blood samples from human healthy donors were obtained with informed consent from Basque Biobank under the approval of Basque Country’s Ethics committee following the Helsinki Convention and all ethical regulations relevant to human research participants were followed.

### Bacterial strains and growth conditions

In this study, *Akkermansia muciniphila* strain ATCC BAA-835 (*A.m*) isolated from human feces was used. *A. muciniphila* was grown under anaerobic conditions in Brain Heart Infusion (BHI) medium at 37 °C for 48 hours. Antibiograms were performed on this strain to find effective antibiotics to use in the assays, showing that *A. muciniphila* ATCC BAA-835 is ampicillin-sensitive at 1 mg/ml. For colon ex vivo experiments, a clinical isolate of *Escherichia coli*, obtained from Marqués de Valdecilla Hospital, was also used. *E. coli* was grown under aerobic conditions in LB broth at 37 °C.

To calculate the multiplicity of infection (MOI), an estimation of bacterial numbers in culture was performed by calculating a correlation equation between optical density at 600 nm and colony-forming units determined by dilution plating. Heat-killed bacteria were obtained by incubating bacteria at 70 °C for 15 min, after which bacterial viability was assessed by plating.

### Exposure to activated macrophages using colon culture ex vivo model

In order to evaluate the effect of *A. muciniphila* within activated macrophages, an animal model of colitis was used, followed by ex vivo colon culture (Fig. 3a). Colon tissues (1 cm) from DSS-induced colitis (2% DSS for 6 days followed by two days of water) C57Bl/6 mice were sampled and placed in cell strainers onto a six-well plate, containing complete DMEM, 20 µg/ml gentamicin and 1 mg/ml ampicillin. After 2 hours of incubation at 37 °C, antibiotics were removed by washing the colon with conditioned DMEM without antibiotics. Colon pieces were then placed into 12-well plates in 2 ml of DMEM with 10% FBS, and some wells were unstimulated or stimulated with 10^6^ CFU per 100 mg tissue with live *A. muciniphila* (*A.m*), heat-killed *A. muciniphila* (Hk*A.m*) or pathogenic *Escherichia coli* 17-7340 (*E. coli*). Plates were incubated at 37 °C during 8-12 h, and subsequently supernatants were collected for cytokine (TNF and IL-10) quantification and the colon pieces for RNA extraction.

### Mammalian cell culture

Bone marrow-derived macrophages (BMMs) were generated from 8–12 week-old C57Bl/6. In detail, bone marrow cells were flushed out from clean femurs with 5 ml of DMEM and tibias, filtered through a 70 µm-nylon mesh (Thermo Fisher Scientific), and centrifuged at 400 g for 5 min. Red blood cells were removed using 4 ml of ACK lysis buffer for 5 min at room temperature, and the remaining cells were incubated and plated in 100 mm × 15 mm size Petri dishes at 37 °C for 6 days in DMEM with 10% FBS and 1% penicillin–streptomycin supplemented with 30 ng/ml of M-CSF (Miltenyi Biotec).

Healthy blood donor’s buffy coats were used to isolate monocytes. First, blood cell suspensions were mixed with up to 35 ml with 2 mM EDTA PBS medium, placed onto a 15 ml Ficoll layer, and centrifuged at 400 × *g* for 30 min without brake. The layer corresponding to peripheral blood monocytic cells was collected, washed twice with 2 mM EDTA PBS medium, and monocytes were positively selected using human CD14 microbeads (Miltenyi Biotec) and manual separators for magnetic cell isolation. For this purpose, cells were resuspended in 20 μl of CD14 microbeads and 80 μl of 2 mM EDTA-0.5% BSA-PBS medium per 10^7^ cells for 15 minutes at 4 °C, washed, and centrifuged. The cells were then resuspended in 500 μl of medium, transferred to LS columns placed in manual separators with a magnetic field, and washed three times. The columns were removed from the separator, and labeled cells were flushed out with 1 ml of medium.

### Antibiotic-protection assays

The ability of *A. muciniphila* to survive inside mammalian macrophages and human monocytes was assessed by antibiotic-protection assays. BMMs were seeded onto 24-well plates at a density of 2 × 10^5^ cells, and 5 × 10^4^ human monocytes were seeded onto 96-well plates. After 24 h, live (*A.m*) or heat-killed *A. muciniphila* (Hk*A.m*) were added at a multiplicity-of-infection (MOI) of 10 in serum- and antibiotic-free DMEM. Infections were first incubated 30 min at 4 °C and then, transferred to an incubator at 37 °C, 5% CO_2_ for 45 min to promote phagocytosis. After the incubation period, extracellular bacteria were washed away with pre-warmed PBS, and those remaining were eliminated after 1 h incubation in DMEM supplemented with 10% serum and antibiotics (1% penicillin-streptomycin and 1 mg/ml ampicillin). Then, phagocytes were lysed at different time points (T1, T4, and T24) in DMEM/0.1% Triton X-100, and lysates were plated onto BHI agar plates for CFU quantification. Samples from the supernatant were also seeded to test the absence of extracellular bacteria after the incubation with the antibiotics. Unstimulated (U) group was used as control.

To assess whether *A. muciniphila* can extrude from phagocytes, bacteria were added at an MOI of 10, and phagocytosis was promoted as previously detailed. Extracellular bacteria were eliminated by extensive washing and the addition of antibiotics. After one (T1) or four hours (T4) with antibiotics, the medium was replaced with antibiotic-free DMEM and cultures were incubated at 37 °C, 5% CO_2_. After 24 h, bacteria from the supernatant and the cell lysates were plated for CFU quantification.

### Bacterial viability staining

To confirm the presence of living bacteria inside phagocytes, intracellular bacteria were analyzed by confocal fluorescence microscopy, as previously reported^6^. In brief, 2 × 10^5^ BMMs were stimulated with *A. muciniphila* with an MOI of 10 to promote phagocytosis, as detailed for the antibiotic-protection assay. At T4, cells were fixed in 4% paraformaldehyde/PBS for 20 min at room temperature. Samples were washed three times with 0.85% NaCl, and the presence of live or dead bacteria inside phagocytes was assessed using the LIVE/DEAD™ *Bac*Light™ Bacterial Viability Kit (Invitrogen), staining cells 15 min at dark conditions. This kit consists of two fluorophores, SYTO 9 and propidium iodide (PI). While SYTO 9 is a green dye that stains bacteria even with intact membranes (LIVE), PI is a red fluorophore used for staining damaged bacteria membranes (DEAD). After removing dye excess, cell nuclei were stained with DAPI and coverslips were mounted with Prolong Gold Anti-fade mounting reagent (Thermo Fisher Scientific). Images were obtained using a Zeiss LSM 880 Confocal System.

### Trained immunity induction assays

BMMs were seeded at 2 × 10^5^ cells/well onto 24-well plates in DMEM supplemented with 10% FBS and 1% P/S. For TNF, IL-10, ROS, lactate, and gene expression measurements 10^6^ cells/well were seeded onto 6-well plates. After 2 hours, cells were washed, and the first stimulation with live (*A.m*) or heat-killed (Hk*A.m*) *A. muciniphila* with fresh DMEM was added at an MOI of 10 to certain experimental groups (*A.m*-*A.m* and Hk*A.m*-*A.m*) and medium alone (U) for unstimulated wells (U-*A.m* and U-U) for 24 hours. Then, cells were washed and incubated in complete DMEM plus 1 mg/ml ampicillin for 24 hours. Macrophages were washed again, and the second stimulation with fresh medium was applied. *A. muciniphila* at an MOI of 10 was added to the *A.m*-*A.m* and Hk*A.m*-*A.m* groups and medium alone for unstimulated wells (U-U). At this point, an intracellular survival assay was performed at different intervals (T1, T4, and T24). Supernatants and cells were collected after 24 hours for further analysis. U-U was used as a control group.

Human monocytes were seeded at a density of 2 × 10^5^ cells/well onto 96-well plates in RPMI supplemented with 10% FBS, 1% P/S, 2 mM of L-glutamine, and 1 mM of sodium pyruvate. For cytokine quantification, 10^6^ cells/well were seeded onto 12-well plates. We followed the same protocol as described with murine macrophages, except for a 6-day resting period between stimuli.

### RNA extraction and real-time PCR

For gene expression analysis, RNA was extracted from murine BMMs, human monocytes, or colon tissues with the Macherey-Nagel™ NucleoSpin™ RNA extraction kit. Only colon tissues were previously treated in 500 μl of TRIzol, and the phases were separated by adding 100 μl of chloroform and centrifuging for 15 min, 4 °C at 10,000 × g. Later, the aqueous phase was transferred to a new tube, mixed with 350 μl of isopropanol, and centrifuged for 4 min, 4 °C at 12,000 × g. The pellets were mixed with 75% ethanol and transferred to Macherey-Nagel™ NucleoSpin™ RNA extraction kit columns. Samples were eluted in 50 μl of nuclease-free water, and cDNA was synthesized with M-MLV Reverse Transcriptase (Thermo Fisher). Gene amplification and detection were performed with QuantStudio 6 Flex Real-Time PCR system in 384-well format, using PerfeCTa SYBR Green SuperMix reagent (Quantabio) and specific primers at their annealing temperature (Table 1). Relative quantification was carried out by measuring the expression of target genes relative to the housekeeping gene *Rpl19* for mice and actin in human monocytes (ΔΔCt method). Gene expression in antibiotic-protection assays was expressed relatively to the mean of unstimulated BMMs (U), in innate immune memory assays to unstimulated murine BMMs or human monocytes in both stimulus (U-U) and in colon ex vivo assays to unstimulated colon tissue (U).

**Table 1:** Forward and reverse primer sequences (5’–3’) and their annealing temperature (T^a^) for gene expression analysis

Gene	Forward (F) and reverse (R) primer sequences	T^a^ (°C)
*Cxcl3*	F:ACCCAGACAGAAGTCATAGCC	60
	R:AGACACATCCAGACACCGTT	
*Il1β*	F: ACACTCCTTAGTCCTCGGCCA	60
	R: CCATCAGAGGCAAGGAGGAA	
*Il6*	F:ACCACGGCCTTCCCTACTTCAC	60
	R:TTCTCATTTCCACGATTTCCCAG	
*Il10*	F:TGGCCCAGAAATCAAGGAGC	60
	R:CAGCAGACTCAATACACACT	
*Il12b*	F:CAGCAAAGCAAGATGTGTCCT	60
	R:TCCATGTCTCTGGTCTGAGGT	
*Rpl19*	F: GACCAAGGAAGCACGAAAGC	60
	R: CAGGCCGCTATGTACAGACA	
*Tlr2*	F:GCAGAATCAATACAATAGAGGGAGAC	60
	R:AAGTGAAGAGTCAGGTGATGGATGTC	
*Tlr4*	F:CGAATGTCTCTGGCAGGTGTA	60
	R:CAAGGGATAAGAACGCTGAGA	
*Tnf*	F: AGCCCACGTCGTAGCAAACCAC	60
	R: ATCGGCTGGCACCACTAGTTGGT	
*ACTB*	F: GCACAGAGCCTCGCCTT	60
	R: CCTTGCACATGCCGGAG	
*IL10*	F: GCTGTTGAGCTGTTTTCCCTG	60
	R: TCCGAGACACTGGAAGGTGA	
Conditions	Hold cycle 95 °C for 2.30’, and then 40× (95 °C for 15”, 60 °C for 1´)	
Reagents	PerfeCTa SYBR® Green SuperMix (Quantabio)	
Instrument	QuantStudio 6 Flex Real-Time PCR System	
Expression	Normalized to *Rpl19* (mouse) and *ACTB* (human) housekeeping genes using the Pfaffl equation and expressed relative to the mean of a relevant control group	

### RNA-sequencing transcriptomics

Following the trained immunity induction assay described above with live *A. muciniphila*, RNA from unstimulated, acutely stimulated, and trained and restimulated BMM was extracted from 1 × 10^6^ cells per condition in triplicates, 16 h after the second stimulus. The quantity and quality of the RNAs were evaluated using Qubit RNA HS Assay Kit (Thermo Fisher Scientific) and Agilent Bioanalyzer RNA 6000 Nano Chips (Agilent Technologies), respectively. Sequencing libraries were prepared following “TruSeq Stranded mRNA Sample Preparation Guide (Part # 15031058 Rev. E)” using the “TruSeq® Stranded mRNA Library Prep” kit (Illumina Inc.) and TruSeq RNA Single Indexes (Illumina Inc.). In brief, starting from 450 ng of total RNA, mRNA was purified, fragmented, and primed for cDNA synthesis. The first cDNA strand was synthesized with SuperScript-II Reverse Transcriptase (Thermo Fisher Scientific) for 10 min at 25 °C, 15 min at 42 °C, 15 min at 70 °C and pause at 4 °C. The second strand of cDNA was synthesized with Illumina reagents at 16 °C for 1 hour. Then, A-tailing and adaptor ligation were performed. Finally, enrichment of libraries was achieved by PCR (30 sec at 98 °C; 15 cycles of 10 sec at 98 °C, 30 sec at 60 °C, 30 sec at 72 °C; 5 min at 72 °C and pause at 4 °C). Afterwards, libraries were visualized on an Agilent 2100 Bioanalyzer using the Agilent High Sensitivity DNA kit (Agilent Technologies) and quantified using the Qubit dsDNA HS DNA Kit (Thermo Fisher Scientific). The libraries were sequenced in a HiSeq2500 (Illumina Inc.) to obtain at least 11 million 50-nucleotides single-reads per library.

Reads were aligned to the mouse reference genome (mm10) using STAR (version_2.7.5c) in two-pass mode following STAR best practices and recommendations^38^. The quality of the data was evaluated using STAR (version 2.7.5c)^38^ and samtools (version 1.15)^39,40^. PCR duplicates were removed from aligned bam files using samtools (version 1.15)^40^. Read counts were extracted from the aligned bam files using subread’s FeatureCounts (version 2.0.3)^41^. Normalization of read counts for analysis was done according to EdgeR recommendations using the Ratio of the Variance method, which accounts for inter-sample variance, and the differential expression analysis of the normalized read counts between the sample groups was performed following best practices and recommendations of EdgeR^42–44^ and Limma^45^ on the R environment (version 3.6.0). Pathway over-representation analyses were performed as implemented in KEGG (KEGGREST version 1.24.1) R package^46^, selecting only DEGs with FDR < 0.05. All the codes used for data analysis are available upon request.

### Quantification of intracellular ROS levels

To quantify intracellular ROS superoxide production in the mitochondrial matrix, cells were incubated with 2.5 µM MitoSOX (Red Mitochondrial Superoxide Indicator) (ThermoFisher) in PBS with Ca^2+^ and Mg^2+^ for 10 min at 37 °C in the dark. The relative fluorescence units (RFU) of mitochondrial ROS, which excite at 510 nm and emit at 580 nm, were measured by FlSpectraMax® M3 Multi-Mode Microplate Reader from Molecular Devices. Protein normalization was performed by extracting proteins with RIPA buffer from each well and measuring protein with the Pierce™ Rapid Gold BCA Protein Assay Kit (ThermoFisher) at 480 nm.

### Lactate measurement

Lactate production by BMM macrophages was measured in culture supernatants with the Lactate-GIo Assay kit from Promega following the manufacturer´s protocol. The protocol involves combining 50 μl of diluted supernatant (1/20) or standard curve points with 50 μl of lactate detection reagent. Subsequently, the mixture is incubated on a plate for 60 minutes at room temperature. A 12-point standard curve was made by creating ½ dilutions starting from 200 μM of lactate to 0.097 μM. Bioluminescent NADH detection obtained from lactate oxidation was recorded using a Veritas Microplate Luminometer (Turner BioSystems, Sunnyvale, CA, USA).

### Metabolic profiling

The extracellular acidification rates (ECAR) and real-time oxygen consumption rates (OCR) were assessed using the Seahorse XF-24 Analyzer (Seahorse Bioscience). A total of 300,000 cells/well for the Unst-Unst and Unst-Akk groups, or 200,000 cells/well for the HAkk-Akk and Akk-Akk groups in a volume of 100 µL were seeded in Agilent seahorse FX24 cell culture microplates. The plates were incubated at room temperature for 30 minutes before being incubated for 4 h at 37 °C, 5% CO_2_. Following the incubation, fresh medium (DMEM, Thermo Fischer Scientific) was added up to a volume of 500 µL. The HAkk-Akk and Akk-Akk groups were stimulated with either heat-killed (70 °C, 30 minutes) or live *A. muciniphila* at a MOI 10 and incubated overnight. In the morning, all plates were washed twice, and the medium was changed to DMEM supplemented with 10% FBS, 1% Penicillin-Streptomycin, and 1:100 Ampicilin. The next day, all groups except for Unst-Unst were stimulated with live *A. muciniphila* at a MO1 10 and incubated overnight. After treatment, the cells were washed in XF assay media (Thermo Fischer Scientific) supplemented with glucose 25 mM, Pyruvate 10 mM, and L-glutamine 4 mM and adjusted to pH 7.4. The cells were then maintained in 500 μL/well of XF assay media at 37 °C in a non-CO_2_ incubator for 1 h. During the incubation time, oligomycin, carbonyl cyanide-4 (trifluoromethoxy) phenylhydrazone (FCCP), and rotenone and antimycin A (Rot/AA) were loaded for the Mito Stress Test. Rot/AA and 2-deoxy-glucose (2-DG) were loaded for the Glycolytic Rate Test in XF assay media into the injection ports in the XF-24 sensor cartridge following manufacturer recommendations. All reagents were purchased from Thermo Fischer Scientific, except for 2-DG, which was from Sigma Aldrich. The measurements were normalized by protein content (BCA assay, Thermo Fischer Scientific). The dataset was analyzed by XF-24 software, and the energy plots (cell energy phenotype test) were generated by following the manufacturer’s guidelines and instructions (Seahorse, Agilent Technologies).

### ELISA

TNF, IL-6, and IL-10 levels were determined in cell supernatants using mouse and human TNF ELISA set II (BD OptEIA), mouse IL-10 ELISA set (BD OptEIA) and human IL-6 ELISA set (BD OptEIA), according to the manufacturer’s recommendations.

### Statistics and reproducibility

Statistical analyses were performed using the GraphPad Prism software (GraphPad Software Inc.). At least three biological replicates were carried out to measure each parameter in each experimental condition. For antibiotic protection and mouse BMMs and human monocytes training assays, the significance was assessed by ordinary one-way ANOVA for 3 or more groups (Tukey post hoc test). To determine the significance of *A. muciniphila* to extrude from macrophages, a 2-way ANOVA was performed (Sidak post hoc test). For colon ex vivo assays, one-way ANOVA and mixed-effects analysis for repeated measures were used (Tukey post hoc test). A *p* values ≤ 0.05 was considered significant and is marked as follows: *p < 0.05; **p < 0.01; ***p ≤ 0.001; ****p < 0.0001.

### Reporting summary

Further information on research design is available in the Nature Portfolio Reporting Summary linked to this article.

### Supplementary information

Supplementary Information

Description of Additional Supplementary Files

Supplementary Data 1

Supplementary Data 2

Supplementary data 3

Reporting Summary

## Data Availability

The data supporting the findings of this study are available within the article and its supplementary materials. The source data underlying Figs. 2 and 5 can be found in the Supplementary data sets 1 and 2. The accession number for the RNAseq data is NCBI GEO: GSE233857. The source data underlying each figure can be found in Supplementary Data 3.
