# Supplementary material for: Akkermansia muciniphila-induced trained immune phenotype increases bacterial intracellular survival and attenuates inflammation
Source: Commun Biol. 2024 Feb 16;7:192. doi: 10.1038/s42003-024-05867-6 (PMC10873422; doi:10.1038/s42003-024-05867-6)
Supplement: Supplementary file 1 — Supplementary Information [file 42003_2024_5867_MOESM1_ESM.pdf]

SUPPLEMENTARY FIGURE 1

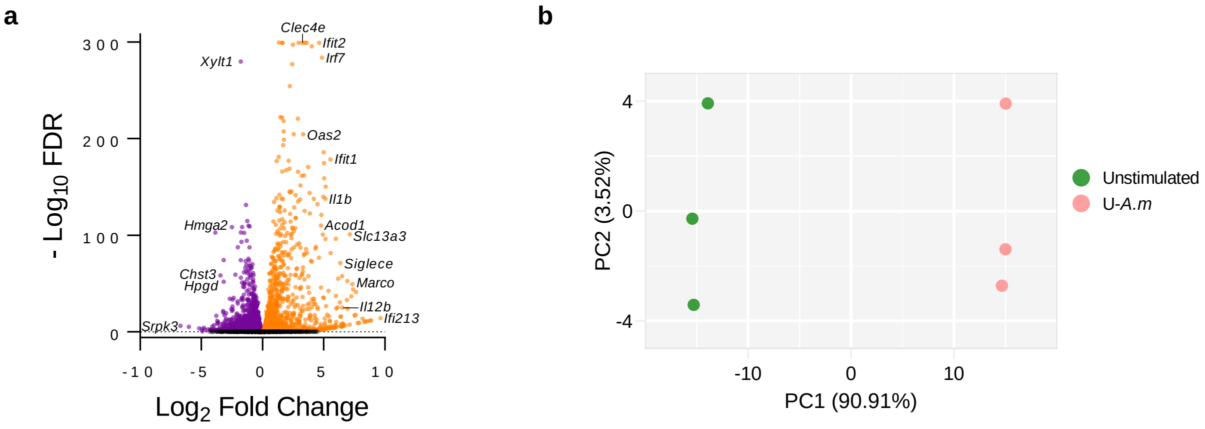

Supplementary Fig. 1 Transcriptional response of live *Akkermansia muciniphila*-stimulated BMMs. a Volcano plot representing differentially expressed genes of acutely stimulated macrophages compared to unstimulated cells, analyzed by RNAseq (n=3). **b** Principal component analysis (PCA) of the transcriptome of unstimulated and acutely stimulated macrophages.
