# Supplementary material for: Akkermansia muciniphila-induced trained immune phenotype increases bacterial intracellular survival and attenuates inflammation
Source: Commun Biol. 2024 Feb 16;7:192. doi: 10.1038/s42003-024-05867-6 (PMC10873422; doi:10.1038/s42003-024-05867-6)
Supplement: Supplementary file 2 — Description of Additional Supplementary Files [file 42003_2024_5867_MOESM2_ESM.pdf]

Description of Additional Supplementary Files

File name: Supplementary Data 1
Description: KEGG enrichment of ACUTE vs. UU UPREGULATED DEGs.

File name: Supplementary Data 2
Description: KEGG enrichment of TRAINED vs. ACUTE UPREGULATED DEGs.

File name: Supplementary Data 3
Description: Data used to run statistical analyses.
