# Supplementary material for: Akkermansia muciniphila-induced trained immune phenotype increases bacterial intracellular survival and attenuates inflammation
Source: Commun Biol. 2024 Feb 16;7:192. doi: 10.1038/s42003-024-05867-6 (PMC10873422; doi:10.1038/s42003-024-05867-6)
Supplement: Supplementary file 3 — Supplementary Data 1 [file 42003_2024_5867_MOESM3_ESM.pdf]

SUPPLEMENTARY TABLE 1. KEGG enrichment of ACUTE vs. UU UPREGULATED DEGs

ID	Description	GeneRatio	BgRatio	qvalue
mmu04621	NOD-like receptor signaling pathway	78/1002	213/8983	9.55E-21
mmu05169	Epstein-Barr virus infection	81/1002	231/8983	1.75E-20
mmu05164	Influenza A	67/1002	173/8983	1.14E-19
mmu05140	Leishmaniasis	39/1002	70/8983	4.43E-18
mmu04668	TNF signaling pathway	50/1002	113/8983	1.16E-17
mmu05417	Lipid and atherosclerosis	66/1002	216/8983	1.54E-13
mmu05167	Kaposi sarcoma-associated herpesvirus infection	66/1002	224/8983	9.39E-13
mmu05152	Tuberculosis	57/1002	180/8983	1.65E-12
mmu05145	Toxoplasmosis	42/1002	110/8983	2.58E-12
mmu04625	C-type lectin receptor signaling pathway	41/1002	112/8983	2.36E-11
mmu05162	Measles	48/1002	146/8983	2.56E-11
mmu05020	Prion disease	70/1002	268/8983	5.84E-11
mmu05012	Parkinson disease	69/1002	264/8983	7.50E-11
mmu04062	Chemokine signaling pathway	55/1002	192/8983	2.39E-10
mmu04145	Phagosome	52/1002	182/8983	8.99E-10
mmu04620	Toll-like receptor signaling pathway	35/1002	100/8983	2.70E-09
mmu05133	Pertussis	30/1002	77/8983	2.70E-09
mmu04064	NF-kappa B signaling pathway	36/1002	105/8983	2.74E-09
mmu05134	Legionellosis	26/1002	61/8983	3.62E-09
mmu04380	Osteoclast differentiation	40/1002	128/8983	6.08E-09
mmu04217	Necroptosis	49/1002	176/8983	6.19E-09
mmu05208	Chemical carcinogenesis - reactive oxygen species	57/1002	222/8983	7.11E-09
mmu05170	Human immunodeficiency virus 1 infection	60/1002	240/8983	7.49E-09
mmu03050	Proteasome	22/1002	47/8983	7.50E-09
mmu05323	Rheumatoid arthritis	31/1002	87/8983	1.20E-08
mmu05163	Human cytomegalovirus infection	62/1002	256/8983	1.31E-08
mmu05418	Fluid shear stress and atherosclerosis	43/1002	148/8983	1.31E-08
mmu04657	IL-17 signaling pathway	32/1002	93/8983	1.60E-08
mmu04141	Protein processing in endoplasmic reticulum	47/1002	172/8983	1.97E-08
mmu05161	Hepatitis B	45/1002	163/8983	2.93E-08
mmu05132	Salmonella infection	60/1002	253/8983	4.90E-08
mmu04622	RIG-I-like receptor signaling pathway	26/1002	70/8983	7.30E-08
mmu04612	Antigen processing and presentation	30/1002	90/8983	9.79E-08
mmu05321	Inflammatory bowel disease	24/1002	62/8983	9.79E-08
mmu05160	Hepatitis C	44/1002	165/8983	1.18E-07
mmu05010	Alzheimer disease	79/1002	383/8983	1.60E-07
mmu05022	Pathways of neurodegeneration - multiple diseases	92/1002	471/8983	1.75E-07
mmu04659	Th17 cell differentiation	32/1002	105/8983	3.31E-07
mmu05168	Herpes simplex virus 1 infection	89/1002	459/8983	3.98E-07
mmu05166	Human T-cell leukemia virus 1 infection	57/1002	250/8983	3.98E-07
mmu04658	Th1 and Th2 cell differentiation	28/1002	88/8983	7.31E-07
mmu05416	Viral myocarditis	28/1002	88/8983	7.31E-07
mmu04932	Non-alcoholic fatty liver disease	40/1002	156/8983	1.34E-06
mmu04060	Cytokine-cytokine receptor interaction	62/1002	292/8983	1.47E-06
mmu05135	Yersinia infection	36/1002	134/8983	1.47E-06
mmu05332	Graft-versus-host disease	22/1002	63/8983	2.40E-06
mmu04940	Type I diabetes mellitus	23/1002	70/8983	4.48E-06
mmu05014	Amyotrophic lateral sclerosis	72/1002	369/8983	4.80E-06
mmu04672	Intestinal immune network for IgA production	17/1002	43/8983	6.08E-06
mmu05171	Coronavirus disease - COVID-19	53/1002	247/8983	6.87E-06
mmu05203	Viral carcinogenesis	50/1002	229/8983	7.80E-06
mmu04514	Cell adhesion molecules	41/1002	174/8983	8.64E-06
mmu04623	Cytosolic DNA-sensing pathway	21/1002	63/8983	8.83E-06
mmu05330	Allograft rejection	21/1002	63/8983	8.83E-06
mmu05016	Huntington disease	60/1002	302/8983	1.86E-05
mmu05142	Chagas disease	28/1002	103/8983	1.88E-05
mmu05415	Diabetic cardiomyopathy	46/1002	211/8983	1.88E-05
mmu04142	Lysosome	33/1002	135/8983	3.31E-05
mmu04061	Viral protein interaction with cytokine and cytokine receptor	26/1002	95/8983	3.37E-05
mmu04210	Apoptosis	33/1002	136/8983	3.79E-05
mmu04216	Ferroptosis	15/1002	40/8983	4.38E-05
mmu04630	JAK-STAT signaling pathway	38/1002	168/8983	4.69E-05
mmu04666	Fc gamma R-mediated phagocytosis	25/1002	92/8983	5.35E-05
mmu05235	PD-L1 expression and PD-1 checkpoint pathway in cancer	23/1002	88/8983	0.000222248
mmu00020	Citrate cycle (TCA cycle)	12/1002	32/8983	0.000305501

mmu05144	Malaria	17/1002	57/8983	0.000328345
mmu01200	Carbon metabolism	28/1002	121/8983	0.000379352
mmu04670	Leukocyte transendothelial migration	27/1002	118/8983	0.000599574
mmu04640	Hematopoietic cell lineage	23/1002	94/8983	0.000608669
mmu05146	Amoebiasis	25/1002	107/8983	0.000700417
mmu04933	AGE-RAGE signaling pathway in diabetic complications	24/1002	101/8983	0.000700417
mmu01230	Biosynthesis of amino acids	20/1002	79/8983	0.000921473
mmu05205	Proteoglycans in cancer	39/1002	205/8983	0.001584005
mmu05320	Autoimmune thyroid disease	19/1002	79/8983	0.002491186
mmu05310	Asthma	9/1002	25/8983	0.002840503
mmu04662	B cell receptor signaling pathway	19/1002	81/8983	0.003349685
mmu05340	Primary immunodeficiency	11/1002	36/8983	0.003741301
mmu00450	Selenocompound metabolism	7/1002	17/8983	0.004063353
mmu00030	Pentose phosphate pathway	10/1002	33/8983	0.006413027
mmu04144	Endocytosis	46/1002	272/8983	0.00650683
mmu05165	Human papillomavirus infection	58/1002	362/8983	0.006917739
mmu01523	Antifolate resistance	9/1002	29/8983	0.00844862
mmu04722	Neurotrophin signaling pathway	24/1002	121/8983	0.00880773
mmu04926	Relaxin signaling pathway	25/1002	129/8983	0.009992667
mmu04613	Neutrophil extracellular trap formation	36/1002	207/8983	0.010751002
mmu04920	Adipocytokine signaling pathway	16/1002	71/8983	0.010751002
mmu00100	Steroid biosynthesis	7/1002	20/8983	0.010759723
mmu05017	Spinocerebellar ataxia	26/1002	141/8983	0.015870558
mmu04917	Prolactin signaling pathway	16/1002	74/8983	0.015870558
mmu00190	Oxidative phosphorylation	25/1002	135/8983	0.017069648
mmu05100	Bacterial invasion of epithelial cells	16/1002	76/8983	0.020218654
mmu05143	African trypanosomiasis	10/1002	39/8983	0.020392392
mmu05221	Acute myeloid leukemia	15/1002	70/8983	0.020992866
mmu04931	Insulin resistance	21/1002	110/8983	0.021091412
mmu04611	Platelet activation	23/1002	124/8983	0.021338269
mmu04810	Regulation of actin cytoskeleton	36/1002	220/8983	0.025427598
mmu00480	Glutathione metabolism	15/1002	72/8983	0.026108821
mmu01524	Platinum drug resistance	16/1002	80/8983	0.030616951
mmu00565	Ether lipid metabolism	11/1002	48/8983	0.03154134

KEGG enrichment of ACUTE vs. UU DOWNREGULATED DEGs

ID	Description	GeneRatio	BgRatio	qvalue
mmu04110	Cell cycle	34/759	125/8983	1.04E-07
mmu05224	Breast cancer	34/759	147/8983	4.39E-06
mmu01521	EGFR tyrosine kinase inhibitor resistance	23/759	79/8983	4.39E-06
mmu04068	FoxO signaling pathway	31/759	131/8983	4.39E-06
mmu04914	Progesterone-mediated oocyte maturation	25/759	92/8983	4.39E-06
mmu04015	Rap1 signaling pathway	39/759	214/8983	0.000104381
mmu05220	Chronic myeloid leukemia	20/759	76/8983	0.000104381
mmu04010	MAPK signaling pathway	48/759	294/8983	0.000172943
mmu04114	Oocyte meiosis	26/759	121/8983	0.000178403
mmu05214	Glioma	19/759	74/8983	0.000190311
mmu04218	Cellular senescence	34/759	184/8983	0.000208295
mmu04072	Phospholipase D signaling pathway	29/759	149/8983	0.000309083
mmu05226	Gastric cancer	29/759	150/8983	0.00032592
mmu04722	Neurotrophin signaling pathway	25/759	121/8983	0.000351764
mmu05202	Transcriptional misregulation in cancer	38/759	224/8983	0.000361488
mmu04510	Focal adhesion	35/759	201/8983	0.000402933
mmu04014	Ras signaling pathway	39/759	235/8983	0.000418054
mmu05225	Hepatocellular carcinoma	31/759	174/8983	0.000650955
mmu01522	Endocrine resistance	20/759	93/8983	0.000947163
mmu05222	Small cell lung cancer	19/759	93/8983	0.002755376
mmu04115	p53 signaling pathway	16/759	72/8983	0.002949248
mmu04152	AMPK signaling pathway	23/759	126/8983	0.003379552
mmu04070	Phosphatidylinositol signaling system	19/759	96/8983	0.003676213
mmu05215	Prostate cancer	19/759	99/8983	0.005215279
mmu04910	Insulin signaling pathway	24/759	139/8983	0.005215279
mmu05218	Melanoma	15/759	72/8983	0.007027675
mmu05223	Non-small cell lung cancer	15/759	72/8983	0.007027675
mmu05213	Endometrial cancer	13/759	58/8983	0.007027675
mmu04660	T cell receptor signaling pathway	19/759	103/8983	0.007027675
mmu05206	MicroRNAs in cancer	42/759	303/8983	0.007027675

mmu04211	Longevity regulating pathway	17/759	90/8983	0.009377638
mmu05231	Choline metabolism in cancer	18/759	98/8983	0.009377638
mmu05211	Renal cell carcinoma	14/759	68/8983	0.00994929
mmu04150	mTOR signaling pathway	25/759	157/8983	0.009995137
mmu00600	Sphingolipid metabolism	12/759	54/8983	0.010156362
mmu04012	ErbB signaling pathway	16/759	84/8983	0.010259429
mmu04213	Longevity regulating pathway - multiple species	13/759	62/8983	0.010834182
mmu04140	Autophagy - animal	23/759	142/8983	0.010834182
mmu04142	Lysosome	22/759	135/8983	0.012156156
mmu04923	Regulation of lipolysis in adipocytes	12/759	56/8983	0.012372719
mmu04979	Cholesterol metabolism	11/759	49/8983	0.012372719
mmu05210	Colorectal cancer	16/759	88/8983	0.014508889
mmu04710	Circadian rhythm	8/759	30/8983	0.014991699
mmu04917	Prolactin signaling pathway	14/759	74/8983	0.017208494
mmu04935	Growth hormone synthesis, secretion and action	19/759	116/8983	0.019456501
mmu04330	Notch signaling pathway	12/759	60/8983	0.019456501
mmu04928	Parathyroid hormone synthesis, secretion and action	18/759	108/8983	0.019456501
mmu05205	Proteoglycans in cancer	29/759	205/8983	0.019466309
mmu00512	Mucin type O-glycan biosynthesis	8/759	32/8983	0.020334249
mmu04919	Thyroid hormone signaling pathway	19/759	120/8983	0.02570366
mmu04810	Regulation of actin cytoskeleton	30/759	220/8983	0.027120504
mmu00562	Inositol phosphate metabolism	13/759	72/8983	0.030327818
mmu04151	PI3K-Akt signaling pathway	44/759	359/8983	0.032357473
mmu04662	B cell receptor signaling pathway	14/759	81/8983	0.032357473
mmu03030	DNA replication	8/759	35/8983	0.032357473
mmu04144	Endocytosis	35/759	272/8983	0.032592311
mmu04664	Fc epsilon RI signaling pathway	12/759	66/8983	0.034893438
mmu05135	Yersinia infection	20/759	134/8983	0.034893438
mmu04934	Cushing syndrome	23/759	162/8983	0.036944068
