# Supplementary material for: Akkermansia muciniphila-induced trained immune phenotype increases bacterial intracellular survival and attenuates inflammation
Source: Commun Biol. 2024 Feb 16;7:192. doi: 10.1038/s42003-024-05867-6 (PMC10873422; doi:10.1038/s42003-024-05867-6)
Supplement: Supplementary file 4 — Supplementary Data 2 [file 42003_2024_5867_MOESM4_ESM.pdf]

SUPPLEMENTARY TABLE 2. KEGG enrichment of TRAINED vs. ACUTE UPREGULATED DEGs

ID	Description	GeneRatio	BgRatio	qvalue
mmu00562	Inositol phosphate metabolism	21/783	72/8983	8.43E-05
mmu04062	Chemokine signaling pathway	38/783	192/8983	8.43E-05
mmu04066	HIF-1 signaling pathway	27/783	114/8983	8.43E-05
mmu04919	Thyroid hormone signaling pathway	27/783	120/8983	0.000182435
mmu04070	Phosphatidylinositol signaling system	23/783	96/8983	0.000254453
mmu04010	MAPK signaling pathway	48/783	294/8983	0.000454825
mmu04928	Parathyroid hormone synthesis, secretion and action	24/783	108/8983	0.000454825
mmu04380	Osteoclast differentiation	26/783	128/8983	0.000951656
mmu04371	Apelin signaling pathway	27/783	137/8983	0.00103027
mmu03250	Viral life cycle - HIV-1	16/783	61/8983	0.00103027
mmu05321	Inflammatory bowel disease	16/783	62/8983	0.001110048
mmu05145	Toxoplasmosis	23/783	110/8983	0.001110048
mmu05140	Leishmaniasis	17/783	70/8983	0.001243341
mmu04140	Autophagy - animal	27/783	142/8983	0.001243341
mmu04144	Endocytosis	43/783	272/8983	0.001243341
mmu05162	Measles	27/783	146/8983	0.001853574
mmu04330	Notch signaling pathway	15/783	60/8983	0.001883704
mmu05211	Renal cell carcinoma	16/783	68/8983	0.002323798
mmu05135	Yersinia infection	25/783	134/8983	0.002373035
mmu04659	Th17 cell differentiation	21/783	105/8983	0.002662721
mmu05161	Hepatitis B	28/783	163/8983	0.003876986
mmu04514	Cell adhesion molecules	29/783	174/8983	0.004922584
mmu04662	B cell receptor signaling pathway	17/783	81/8983	0.004955339
mmu04710	Circadian rhythm	9/783	30/8983	0.006953148
mmu05152	Tuberculosis	29/783	180/8983	0.007339639
mmu04666	Fc gamma R-mediated phagocytosis	18/783	92/8983	0.007339639
mmu04672	Intestinal immune network for IgA production	11/783	43/8983	0.007339639
mmu05167	Kaposi sarcoma-associated herpesvirus infection	34/783	224/8983	0.007612089
mmu04936	Alcoholic liver disease	24/783	141/8983	0.008234471
mmu05163	Human cytomegalovirus infection	37/783	256/8983	0.010610239
mmu04922	Glucagon signaling pathway	19/783	104/8983	0.010610239
mmu05166	Human T-cell leukemia virus 1 infection	36/783	250/8983	0.012511474
mmu05417	Lipid and atherosclerosis	32/783	216/8983	0.012939475
mmu05032	Morphine addiction	17/783	91/8983	0.012939475
mmu04068	FoxO signaling pathway	22/783	131/8983	0.012939475
mmu04935	Growth hormone synthesis, secretion and action	20/783	116/8983	0.013999269
mmu04072	Phospholipase D signaling pathway	24/783	149/8983	0.013999269
mmu04810	Regulation of actin cytoskeleton	32/783	220/8983	0.015757117
mmu00310	Lysine degradation	13/783	64/8983	0.017203002
mmu05170	Human immunodeficiency virus 1 infection	34/783	240/8983	0.017203002
mmu05205	Proteoglycans in cancer	30/783	205/8983	0.017203002
mmu04015	Rap1 signaling pathway	31/783	214/8983	0.017203002
mmu04921	Oxytocin signaling pathway	24/783	153/8983	0.017203002
mmu04658	Th1 and Th2 cell differentiation	16/783	88/8983	0.017667554
mmu05235	PD-L1 expression and PD-1 checkpoint pathway in cancer	16/783	88/8983	0.017667554
mmu05164	Influenza A	26/783	173/8983	0.020198967
mmu04620	Toll-like receptor signaling pathway	17/783	100/8983	0.026274467
mmu05169	Epstein-Barr virus infection	32/783	231/8983	0.02655167
mmu00230	Purine metabolism	21/783	134/8983	0.02668961
mmu04925	Aldosterone synthesis and secretion	17/783	102/8983	0.030251597
mmu04640	Hematopoietic cell lineage	16/783	94/8983	0.030263639
mmu04520	Adherens junction	13/783	71/8983	0.032915525
mmu01521	EGFR tyrosine kinase inhibitor resistance	14/783	79/8983	0.032915525
mmu04022	cGMP-PKG signaling pathway	25/783	173/8983	0.03350512
mmu04668	TNF signaling pathway	18/783	113/8983	0.03499474
mmu04910	Insulin signaling pathway	21/783	139/8983	0.03499474
mmu04064	NF-kappa B signaling pathway	17/783	105/8983	0.03499474
mmu05418	Fluid shear stress and atherosclerosis	22/783	148/8983	0.03499474

KEGG enrichment of TRAINED vs. ACUTE DOWNREGULATED DEGs

ID	Description	GeneRatio	BgRatio	qvalue
mmu04142	Lysosome	51/861	135/8983	1.31E-16
mmu05323	Rheumatoid arthritis	30/861	87/8983	1.77E-08
mmu04064	NF-kappa B signaling pathway	30/861	105/8983	1.65E-06
mmu05152	Tuberculosis	42/861	180/8983	1.65E-06

mmu04668	TNF signaling pathway	31/861	113/8983	1.65E-06
mmu04145	Phagosome	42/861	182/8983	1.65E-06
mmu04625	C-type lectin receptor signaling pathway	29/861	112/8983	1.37E-05
mmu04933	AGE-RAGE signaling pathway in diabetic complications	27/861	101/8983	1.50E-05
mmu05417	Lipid and atherosclerosis	44/861	216/8983	2.30E-05
mmu04141	Protein processing in endoplasmic reticulum	37/861	172/8983	3.47E-05
mmu01240	Biosynthesis of cofactors	34/861	152/8983	3.47E-05
mmu04216	Ferroptosis	15/861	40/8983	3.47E-05
mmu05146	Amoebiasis	26/861	107/8983	0.000103971
mmu04721	Synaptic vesicle cycle	21/861	77/8983	0.00011009
mmu04210	Apoptosis	30/861	136/8983	0.000145904
mmu04966	Collecting duct acid secretion	11/861	27/8983	0.000248511
mmu00270	Cysteine and methionine metabolism	16/861	54/8983	0.000362974
mmu05418	Fluid shear stress and atherosclerosis	30/861	148/8983	0.000675909
mmu05140	Leishmaniasis	18/861	70/8983	0.000851527
mmu00970	Aminoacyl-tRNA biosynthesis	17/861	66/8983	0.001246124
mmu05222	Small cell lung cancer	21/861	93/8983	0.001425554
mmu05169	Epstein-Barr virus infection	40/861	231/8983	0.001425554
mmu04510	Focal adhesion	36/861	201/8983	0.001425554
mmu04380	Osteoclast differentiation	26/861	128/8983	0.001425554
mmu01200	Carbon metabolism	25/861	121/8983	0.001425554
mmu04137	Mitophagy - animal	17/861	68/8983	0.001425554
mmu01523	Antifolate resistance	10/861	29/8983	0.0017884
mmu05220	Chronic myeloid leukemia	18/861	76/8983	0.0017884
mmu05142	Chagas disease	22/861	103/8983	0.0017884
mmu04621	NOD-like receptor signaling pathway	37/861	213/8983	0.0017884
mmu05132	Salmonella infection	42/861	253/8983	0.001885973
mmu00480	Glutathione metabolism	17/861	72/8983	0.002421916
mmu04926	Relaxin signaling pathway	25/861	129/8983	0.003088124
mmu05134	Legionellosis	15/861	61/8983	0.00314774
mmu00520	Amino sugar and nucleotide sugar metabolism	13/861	51/8983	0.00497013
mmu04659	Th17 cell differentiation	21/861	105/8983	0.005127371
mmu00511	Other glycan degradation	7/861	18/8983	0.005298943
mmu05205	Proteoglycans in cancer	34/861	205/8983	0.00569681
mmu04722	Neurotrophin signaling pathway	23/861	121/8983	0.00569681
mmu05225	Hepatocellular carcinoma	30/861	174/8983	0.00569681
mmu04657	IL-17 signaling pathway	19/861	93/8983	0.005988897
mmu04640	Hematopoietic cell lineage	19/861	94/8983	0.00668557
mmu00600	Sphingolipid metabolism	13/861	54/8983	0.007187345
mmu04658	Th1 and Th2 cell differentiation	18/861	88/8983	0.007353288
mmu05161	Hepatitis B	28/861	163/8983	0.007767954
mmu00604	Glycosphingolipid biosynthesis - ganglio series	6/861	15/8983	0.00821653
mmu04010	MAPK signaling pathway	44/861	294/8983	0.00821653
mmu04979	Cholesterol metabolism	12/861	49/8983	0.00821653
mmu00532	Glycosaminoglycan biosynthesis - chondroitin sulfate / dermatan s	7/861	20/8983	0.00821653
mmu05012	Parkinson disease	40/861	264/8983	0.009660427
mmu05010	Alzheimer disease	54/861	383/8983	0.009660427
mmu05163	Human cytomegalovirus infection	39/861	256/8983	0.009660427
mmu05022	Pathways of neurodegeneration - multiple diseases	64/861	471/8983	0.009660427
mmu05144	Malaria	13/861	57/8983	0.009660427
mmu04630	JAK-STAT signaling pathway	28/861	168/8983	0.009903793
mmu00531	Glycosaminoglycan degradation	7/861	21/8983	0.009903793
mmu00603	Glycosphingolipid biosynthesis - globo and isoglobo series	6/861	16/8983	0.010091625
mmu04620	Toll-like receptor signaling pathway	19/861	100/8983	0.010268214
mmu05162	Measles	25/861	146/8983	0.010750104
mmu01230	Biosynthesis of amino acids	16/861	79/8983	0.010750104
mmu04014	Ras signaling pathway	36/861	235/8983	0.010935344
mmu00450	Selenocompound metabolism	6/861	17/8983	0.013190094
mmu05208	Chemical carcinogenesis - reactive oxygen species	34/861	222/8983	0.013670198
mmu05165	Human papillomavirus infection	50/861	362/8983	0.016938922
mmu04012	ErbB signaling pathway	16/861	84/8983	0.018694733
mmu05215	Prostate cancer	18/861	99/8983	0.018703058
mmu04015	Rap1 signaling pathway	32/861	214/8983	0.023538563
mmu01521	EGFR tyrosine kinase inhibitor resistance	15/861	79/8983	0.023538563
mmu00620	Pyruvate metabolism	10/861	44/8983	0.023538563
mmu04150	mTOR signaling pathway	25/861	157/8983	0.023538563
mmu04072	Phospholipase D signaling pathway	24/861	149/8983	0.023538563

mmu04931	Insulin resistance	19/861	110/8983	0.024182766
mmu05167	Kaposi sarcoma-associated herpesvirus infection	33/861	224/8983	0.024182766
mmu04140	Autophagy - animal	23/861	142/8983	0.024610038
mmu00020	Citrate cycle (TCA cycle)	8/861	32/8983	0.026092096
mmu00190	Oxidative phosphorylation	22/861	135/8983	0.026092096
mmu04662	B cell receptor signaling pathway	15/861	81/8983	0.026346531
mmu04918	Thyroid hormone synthesis	14/861	74/8983	0.027293498
mmu05231	Choline metabolism in cancer	17/861	98/8983	0.031101816
mmu05212	Pancreatic cancer	14/861	76/8983	0.033550266
