# Supplementary material for: Akkermansia muciniphila-induced trained immune phenotype increases bacterial intracellular survival and attenuates inflammation
Source: Commun Biol. 2024 Feb 16;7:192. doi: 10.1038/s42003-024-05867-6 (PMC10873422; doi:10.1038/s42003-024-05867-6)
Supplement: Supplementary file 6 — Reporting Summary [file 42003_2024_5867_MOESM6_ESM.pdf]

nature portfolio

Corresponding author(s): J Anguita and L Abecia

Last updated by author(s): January 12th, 2024

Reporting Summary

Nature Portfolio wishes to improve the reproducibility of the work that we publish. This form provides structure for consistency and transparency in reporting. For further information on Nature Portfolio policies, see our Editorial Policies and the Editorial Policy Checklist.

Statistics

For all statistical analyses, confirm that the following items are present in the figure legend, table legend, main text, or Methods section.

n/a	Confirmed	
☐	☒	The exact sample size (*n*) for each experimental group/condition, given as a discrete number and unit of measurement
☐	☒	A statement on whether measurements were taken from distinct samples or whether the same sample was measured repeatedly
☐	☒	The statistical test(s) used AND whether they are one- or two-sided<br>*Only common tests should be described solely by name; describe more complex techniques in the Methods section.*
☒	☐	A description of all covariates tested
☐	☒	A description of any assumptions or corrections, such as tests of normality and adjustment for multiple comparisons
☐	☒	A full description of the statistical parameters including central tendency (e.g. means) or other basic estimates (e.g. regression coefficient) AND variation (e.g. standard deviation) or associated estimates of uncertainty (e.g. confidence intervals)
☐	☒	For null hypothesis testing, the test statistic (e.g. *F*, *t*, *r*) with confidence intervals, effect sizes, degrees of freedom and *P* value noted<br>*Give P values as exact values whenever suitable.*
☒	☐	For Bayesian analysis, information on the choice of priors and Markov chain Monte Carlo settings
☒	☐	For hierarchical and complex designs, identification of the appropriate level for tests and full reporting of outcomes
☒	☐	Estimates of effect sizes (e.g. Cohen's *d*, Pearson's *r*), indicating how they were calculated

Our web collection on statistics for biologists contains articles on many of the points above.

Software and code

Policy information about availability of computer code

Data collection	Not software was used
Data analysis	The following open source code was used for the RNAseq analysis:<br>- STAR (version_2.7.5c) in two-pass mode following STAR best practices and recommendations: alignment of reads to the mouse reference genome (mm10) and quality of the data evaluation<br>- Samtools (version 1.15): quality of the data evaluation and removal of PCR duplicates<br>- FeatureCounts (version 2.0.3): extraction of read counts from the aligned bam files<br>- EdgeR and Limma on the R environment (version 3.6.0): Normalization of read counts for analysis using the Ratio of the Variance method and the differential expression analysis of the normalized read counts between the sample groups<br>- KEGG (KEGGREST version 1.24.1) R package: Pathway over-representation analyses<br><br>Graphpad for statistical analysis of experiments data

For manuscripts utilizing custom algorithms or software that are central to the research but not yet described in published literature, software must be made available to editors and reviewers. We strongly encourage code deposition in a community repository (e.g. GitHub). See the Nature Portfolio guidelines for submitting code & software for further information.

Data

Policy information about availability of data

All manuscripts must include a data availability statement. This statement should provide the following information, where applicable:

- Accession codes, unique identifiers, or web links for publicly available datasets
- A description of any restrictions on data availability
- For clinical datasets or third party data, please ensure that the statement adheres to our policy

The data supporting the findings of this study are available within the article and its supplementary materials. The accession number for the RNAseq data is NCBI GEO: GSE233857.

Research involving human participants, their data, or biological material

Policy information about studies with human participants or human data. See also policy information about sex, gender (identity/presentation), and sexual orientation and race, ethnicity and racism.

Reporting on sex and gender	Findings don´t apply to sex or gender
Reporting on race, ethnicity, or other socially relevant groupings	There was not socially relevant grouping
Population characteristics	*Describe the covariate-relevant population characteristics of the human research participants (e.g. age, genotypic information, past and current diagnosis and treatment categories). If you filled out the behavioural & social sciences study design questions and have nothing to add here, write "See above."*
Recruitment	Participants were not actively recruited; instead, blood samples were randomly obtained from donors in the biobank.
Ethics oversight	Blood samples from human healthy donors were obtained from Basque Biobank under the approval of Basque Country's Ethics committee following the Helsinki convention.

Note that full information on the approval of the study protocol must also be provided in the manuscript.

Field-specific reporting

Please select the one below that is the best fit for your research. If you are not sure, read the appropriate sections before making your selection.

☒ Life sciences ☐ Behavioural & social sciences ☐ Ecological, evolutionary & environmental sciences

For a reference copy of the document with all sections, see nature.com/documents/nr-reporting-summary-flat.pdf

Life sciences study design

All studies must disclose on these points even when the disclosure is negative.

Sample size	At least n=3 for all survival experiments. For qPCR some experiments we decided to increase the n number going from 3 to 6. For colon ex vivo experiments 9 mice were used. For RNA-seq analysis, 3 mice per group were used. All n numbers are detailed in the figure legends.
Data exclusions	Data were not excluded.
Replication	Experiments were repeated at least 2 times to make sure we could replicate the experiments.
Randomization	Samples were ramdonly assigned.
Blinding	The investigators refrained from implementing blinding procedures during the experiment due to concerns about potential errors. Nevertheless, the results obtained lacked objectivity as they were derived directly from the assay's objective readout

Reporting for specific materials, systems and methods

We require information from authors about some types of materials, experimental systems and methods used in many studies. Here, indicate whether each material, system or method listed is relevant to your study. If you are not sure if a list item applies to your research, read the appropriate section before selecting a response.

Materials & experimental systems

n/a	Involved in the study
☒	☐ Antibodies
☒	☐ Eukaryotic cell lines
☒	☐ Palaeontology and archaeology
☐	☒ Animals and other organisms
☒	☐ Clinical data
☒	☐ Dual use research of concern
☒	☐ Plants

Methods

n/a	Involved in the study
☒	☐ ChIP-seq
☒	☐ Flow cytometry
☒	☐ MRI-based neuroimaging

Animals and other research organisms

Policy information about studies involving animals; ARRIVE guidelines recommended for reporting animal research, and Sex and Gender in Research

Laboratory animals	8-12 week-old C57Bl/6 mice
Wild animals	The study did not involve wild animals
Reporting on sex	Male and female mice were use without disaggregating data based on sex
Field-collected samples	The study did not involve samples collected from the field
Ethics oversight	All procedures and experiments involving animals were approved by the Animal Research Ethics Board of CIC bioGUNE, according to the guidelines of the European Union Council (Directive 2010/63/EU) and Spanish Government regulations (RD 53/2013).

Note that full information on the approval of the study protocol must also be provided in the manuscript.

Plants

Seed stocks	*Report on the source of all seed stocks or other plant material used. If applicable, state the seed stock centre and catalogue number. If plant specimens were collected from the field, describe the collection location, date and sampling procedures.*
Novel plant genotypes	*Describe the methods by which all novel plant genotypes were produced. This includes those generated by transgenic approaches, gene editing, chemical/radiation-based mutagenesis and hybridization. For transgenic lines, describe the transformation method, the number of independent lines analyzed and the generation upon which experiments were performed. For gene-edited lines, describe the editor used, the endogenous sequence targeted for editing, the targeting guide RNA sequence (if applicable) and how the editor was applied.*
Authentication	*Describe any authentication procedures for each seed stock used or novel genotype generated. Describe any experiments used to assess the effect of a mutation and, where applicable, how potential secondary effects (e.g. second site T-DNA insertions, mosiacism, off-target gene editing) were examined.*
